# Research progress on T-cell responses in *Staphylococcus aureus* infections

**DOI:** 10.3389/fmicb.2026.1815566

**Published:** 2026-06-17

**Authors:** Xupeng Li, Ruidan Wang, Wentong Yan, Jiarui Guan, Haibang Pan, Bo Wang

**Affiliations:** 1 First Clinical Medical College, Gansu University of Chinese Medicine, Lanzhou, Gansu, China; 2 School of Nursing, Gansu University of Chinese Medicine Lanzhou, Gansu, China; 3 Affiliated Hospital of Gansu University of Chinese Medicine, Lanzhou, Gansu, China

**Keywords:** immune evasion, immunoregulatory strategies, *Staphylococcus aureus*, T-cell dysregulation, T-cell response

## Abstract

**Introduction:**

*Staphylococcus aureus* is a common Gram-positive pathogen capable of causing a wide range of severe infections. T cells are key adaptive immune effectors in *S. aureus* infection, and their response patterns and functional states strongly influence bacterial clearance, inflammatory regulation, and clinical outcomes.

**Methods:**

This narrative review summarizes recent evidence on T-cell responses in *S. aureus* infection, with emphasis on major T-cell subsets, context-dependent immune dysregulation, immune evasion, immunopathology, and potential immunoregulatory strategies.

**Results:**

Current evidence indicates that Th1/Th17 responses contribute to antimicrobial defense, whereas regulatory T cells may limit inflammatory damage but may also favor chronic colonization. Across different infection sites and disease stages, CD8+ T cells and innate-like T-cell populations, including γδ T cells, MAIT cells, and NKT cells, display marked functional heterogeneity. Recent advances in single-cell omics, immunometabolism, and immune checkpoint research have further clarified the dynamic changes in T-cell responses during *S. aureus* infection.

**Discussion:**

T-cell responses in *S. aureus* infection are highly context-dependent. A better understanding of these immune programs may inform future prevention and treatment strategies, including vaccines and targeted immunomodulatory interventions.

## 1 Introduction

**Figure 1:**
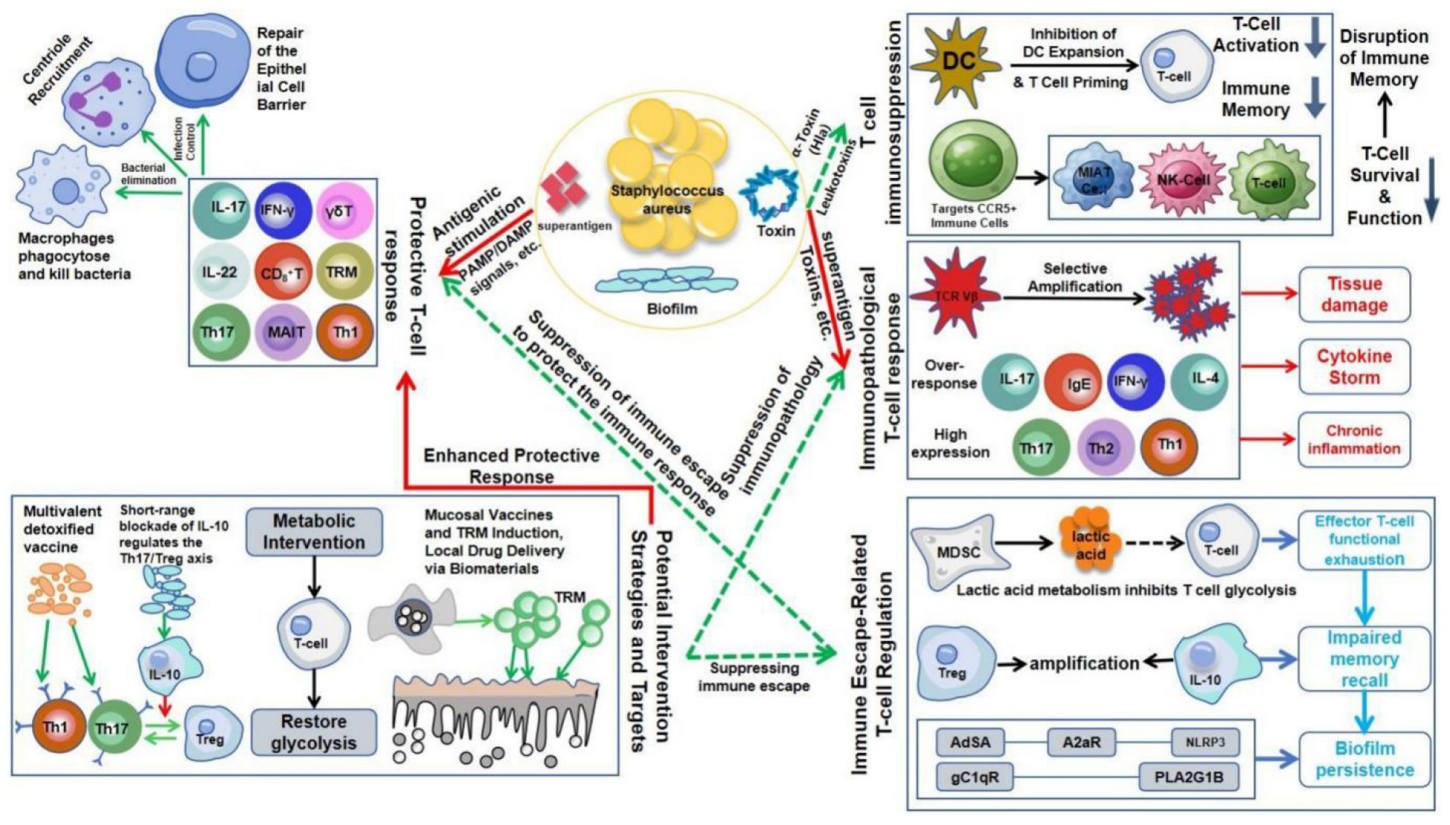
Overview of T-cell responses in *Staphylococcus aureus* infection. Protective programs include Th1/Th17, γδ T, MAIT, and tissue-resident memory T-cells–associated antibacterial immunity, whereas superantigen-driven effector amplification, cytotoxin-mediated immune evasion, and suppressive myeloid/regulatory circuits contribute to immunopathology, chronic persistence, and impaired memory formation. Detailed mechanisms and context-specific interpretations are discussed in the main text.

*Staphylococcus aureus* is a common and highly pathogenic Gram-positive bacterium that causes skin and soft tissue infections, pneumonia, bacteremia, and biofilm-associated infections. Widespread antibiotic use has contributed to the emergence of methicillin-resistant *Staphylococcus aureus* (MRSA), which limits the efficacy of antimicrobial therapy in some settings and increases the risk of prolonged illness and recurrence. These challenges underscore the importance of host immune regulation (Sinclair et al., 2022; Zhong et al., 2023; Scully et al., 2024). T-cells play a central role in the adaptive immune response to *S. aureus* infection, but their functions are strongly context-dependent. In many settings, Th1/Th17-associated responses contribute to bacterial clearance, whereas regulatory or suppressive programs help limit inflammatory injury but may also favor persistence

in selected chronic or biofilm-associated conditions. Further heterogeneity arises from “innate-like” T-cell populations, including CD8+ T-cells, γδ T cells, natural killer T (NKT) cells, and mucosal-associated invariant T (MAIT) cells, whose roles vary across tissues and disease stages. Together, these observations underscore that T-cell immunity in *S. aureus* infection is neither uniform nor static, but shaped by infection site, disease stage, and local immune context (Moreau et al., 2021; Wang et al., 2023; Scully et al., 2024). Recent advances in single-cell omics, immunometabolism, and immune checkpoint research have further revealed how T-cell states change dynamically within infection sites, providing new insight into immune dysregulation and immune evasion. Against this background, the present review focuses on how distinct T-cell programs contribute to protection, immunopathology, immune evasion, and translational intervention strategies in *Staphylococcus aureus* infection. Figure 1 summarizes the overall framework of T-cell responses during *S. aureus* infection and provides a guide to the sections that follow.

## 2 Literature search strategy and review scope

This manuscript was revised and presented as a narrative review. A structured literature search was performed in PubMed for studies published between January 1, 2020 and January 31, 2026, using combinations of the following terms: “*Staphylococcus aureus*”, “MRSA”, “T-cells”, “Th17”, “Treg”, “CD8”, “γδ T cells”, “MAIT”, “NKT”, “TRM”, “immune evasion”, “superantigen”, “alpha-toxin”, “Hla”, “biofilm”, and “vaccine”. Additional papers were identified by screening the reference lists of relevant primary articles and reviews. Literature was selected according to topic relevance, mechanistic value, and representation of major clinical and experimental infection settings. We prioritized English-language primary research articles and high-quality reviews directly related to *Staphylococcus aureus* and T-cell immunity, while excluding publications without clear relevance to T-cell mechanisms, duplicate reports, conference abstracts, and studies lacking accessible full text. As this manuscript is presented as a narrative review rather than a systematic review, a formal PRISMA workflow and study-quality/risk-of-bias assessment were not applied.

### 2.1 Overview of T-cells

T-cells are key effector cells of the adaptive immune system. By recognizing antigens, they contribute to pathogen clearance, inflammatory regulation, and the establishment of immune memory, and thus play a central role in *Staphylococcus aureus* infection (Ferraro et al., 2019; Armentrout et al., 2020). Among these populations, Th1 and Th17 cells, together with their memory counterparts, are considered essential for controlling *S. aureus* infection: Th1 cells produce IFN-γ to enhance macrophage phagocytosis and bactericidal capacity, while Th17 cells promote neutrophil recruitment and the expression of antimicrobial factors in the mucosal barrier by secreting IL-17, forming a key axis for skin and mucosal defense against *S. aureus* (Stockinger and Omenetti, 2017; Ferraro et al., 2019; Armentrout et al., 2020; Clegg et al., 2021). Th2 cells participate in humoral immune responses, while Treg cells limit inflammation and maintain tissue homeostasis through inhibitory factors such as IL-10 (Stockinger and Omenetti, 2017). Additionally, CD8^+^ cytotoxic T-cells (CTLs) mediate killing of infected cells via granzyme and perforin, while secreting IFN-γ and TNF-α to regulate the local immune environment (Koh et al., 2023). Within barrier tissues, γδ T cells, NKT cells, MAIT cells, and tissue-resident memory T cells (TRM) provide rapid responses and sustained local immune surveillance. Notably, TRM make important contributions to early defense against cutaneous *S. aureus* infection (Clegg et al., 2021; Hendriks et al., 2021). Importantly, circulating T-cell readouts in peripheral blood do not necessarily mirror immune events within barrier tissues or infection foci. TRM are maintained in the skin and mucosal surfaces-major sites of *S. aureus* colonization and recurrence-and can be rapidly reactivated to produce IL-17A, IL-22, and IFN-γ, thereby strengthening local antimicrobial defense and reducing reinfection risk (Clegg et al., 2021; Hendriks et al., 2021). Accordingly, blood-based immune signatures in human studies should be interpreted cautiously when inferring local protective immunity. From a translational perspective, promoting or maintaining barrier TRM may be critical for preventing recurrence and persistent colonization (Clegg et al., 2021). This distinction is particularly relevant for skin and mucosal infections, where tissue microenvironmental cues can shape local T-cell differentiation and effector function.

During *S. aureus* infection, T-cell responses exhibit time-dependent, phased changes. In the acute phase, antigen-presenting cells induce a predominantly Th1/Th17-mediated response, enhancing neutrophil and macrophage function while promoting rapid clearance through IFN-γ and IL-17 (Stockinger and Omenetti, 2017; Ferraro et al., 2019; Clegg et al., 2021). However, when the Th17/IL-17 axis remains persistently activated, inflammation may fail to resolve promptly, potentially causing tissue damage and increasing the risk of chronic inflammation (Stockinger and Omenetti, 2017; Bartsch et al., 2022). In recurrent, persistent, or biofilm-associated infections, T-cell function undergoes further dysregulation: sustained Th1/Th17 responses maintain local inflammation, while *S. aureus* utilizes virulence factors and metabolic adaptations to upregulate IL-10 and promote Treg enrichment. This shifts the microenvironment toward immunosuppression, diminishing effector T-cell activity and thereby creating conditions for persistent infection or reactivation (Armentrout et al., 2020; Clegg et al., 2021, 2025). Thus, protective immunity, immunopathology, and immune evasion should be viewed as dynamically shifting states along an acute-to-chronic disease continuum. Accordingly, Th1/Th17 programs tend to be most protective early in acute infection, whereas their persistence or dysregulation during chronic/recurrent or biofilm-associated disease can shift from bacterial control toward immunopathology and immune evasion.

Traditional Th1/Th2/Th17/Treg classification is largely based on relatively stable phenotypes induced by specific cytokines *in vitro*, and therefore does not fully capture the continuum and plasticity of T-cell states *in vivo* across different tissues and stages of infection. Current evidence suggests that the strength and kinetics of T-cell receptor (TCR) signaling, together with the metabolic shift from oxidative phosphorylation to glycolysis in effector T-cells, jointly influence differentiation toward effector or memory lineages. In addition, the same T-cell clone may display markedly different transcriptional states across tissues or disease stages and may undergo reversible lineage reprogramming in response to microenvironmental cues. For example, Th17 cells can undergo T-bet–dependent conversion to a Th1-like phenotype and thereby acquire enhanced effector functions during *S. aureus* infection (Bartsch et al., 2022). Single-cell transcriptomic studies further highlight the marked heterogeneity of T-cell populations within infection foci: effector CD4+ T-cells often co-express multiple classical lineage markers, whereas regulatory and memory T-cell subsets vary substantially with infection stage, bacterial strain, and local microenvironment (Clegg et al., 2021, 2025; Kak et al., 2025). In addition, immune checkpoint pathways shape subsequent T-cell responses. For instance, the PD-1/PD-L1 axis can limit excessive activation by resetting the balance between co-stimulatory and inhibitory signaling and may contribute to the late-stage immune regulatory state during infection (Mazerolles, 2024). Accordingly, within the pathological context of *S. aureus* infection, interpreting T-cell responses in functional terms—such as inflammatory amplification, pathogen clearance, tissue damage and repair, and effector dysfunction—may be more informative than relying solely on static subset labels (Armentrout et al., 2020; Clegg et al., 2021).

### 2.2 Mechanisms of action of major T-cell subpopulations in *Staphylococcus aureus* infection

Based on TCR expression and function, T-cells are broadly divided into αβ T-cells expressing αβ TCR and γδ T cells expressing γδ TCR (Sun et al., 2023). The former group primarily includes CD4+ helper T-cells (such as Th1, Th2, Th17, and Tfh cells), CD4+ regulatory T-cells (Treg cells), and CD8+ cytotoxic T-cells (CTLs), which are mainly responsible for effector cytokine production, immune suppression and tolerance maintenance, and clearance of infected cells together with the establishment of immune memory (Dikiy and Rudensky, 2023; Sun et al., 2023; Peng et al., 2024; Sumida et al., 2024; Wang et al., 2025). The latter category comprises γδ T cells (such as the Vδ1 and Vδ2 subsets) and “innate-like” T cells including NKT and MAIT cells. By recognizing lipid or metabolite antigens presented through non-classical pathways, these cells can rapidly produce large amounts of cytokines, thereby contributing to barrier defense and bridging innate and adaptive immunity (Hackstein and Klenerman, 2023; Sun et al., 2023; McWilliam and Villadangos, 2024; Salou et al., 2025). In *S. aureus* infections, these T-cell subsets do not contribute uniformly across disease phenotypes. Instead, they exhibit distinct functional response patterns depending on the site of infection, stage of disease progression, and pathogen load, as shown in Table 1.

**Table 1:** Major T-cell subsets, dominant effector molecules, principal disease settings, key functions, and predominant evidence source in *S. aureus* infection.

T-cell subsets	Major effector molecules	Principal infection settings	Key features	Predominant evidence source
Th1	IFN-γ, TNF-α	Invasive pneumonia, bacteremia, deep abscess	Promotes macrophage activation and bacterial clearance; Excessive activation may cause tissue damage/systemic inflammation.	Th1 → Mixed (mouse-dominant for mechanism; limited human observational support)
Th17	IL-17A/F, IL-22	Skin and mucosal infections; abscess containment; barrier repair	Promotes neutrophil recruitment and antimicrobial peptide expression; maintains epithelial barrier integrity; sustained activation may contribute to inflammatory tissue damage; exhibits plasticity toward Th1-like phenotypes	Th17 → mixed
Treg	IL-10, TGF-β	Chronic prosthetic joint infection, biofilm-associated infection, recovery phase of septic arthritis	Suppresses excessive inflammation and promotes tissue repair; excessive suppression weakens antimicrobial effects and facilitates colonization/escape.	Treg → Mixed
CD8^+^ T-cells	IFN-γ, Perforin, Granzyme	Influenza complicated by *S. aureus* pneumonia and systemic infection	Clear infected cells and assist in controlling pathogens; may exacerbate tissue damage in viral-bacterial co-infections.	CD8+ T-cells → Mostly mouse models
γδ T cells	IL-17A, IL-22	Skin, breast, and mucosal surface infections	Early rapid production of IL-17 rapidly recruits neutrophils; excessive levels can amplify local inflammation.	γδ T cells → mostly mouse models
MAIT	IFN-γ, IL-17, TNF-α	Bloodstream infection, liver colonization, and other tissue colonization	Identifies bacterial metabolites and participates in early defense; chronic stimulation can lead to functional exhaustion.	MAIT → Human/*in vitro*-dominant
NKT	IFN-γ, IL-4, IL-17	Pulmonary infection, systemic inflammation	Rapidly secretes cytokines to regulate innate/adaptive immunity; When imbalanced, may promote inflammation or suppress immunity.	NKT → Mixed (limited human evidence)

#### 2.2.1 Mechanism of CD4+ helper T-cells in *Staphylococcus aureus* infection

Mechanistic studies in murine models support an important role for CD4^+^ T-cells in host defense against *Staphylococcus aureus*, whereas evidence from human studies has largely been observational and correlative. In *S. aureus* infections, CD4^+^ helper T-cells exhibit diverse functional states across different sites and disease stages, characterized by either antimicrobial defense or inflammatory amplification. Regarding local barriers, *S. aureus*-specific CD4^+^ tissue-resident memory T-cells enriched in the skin of healthy individuals rapidly produce IL-17A, IL-22, and IFN-γ upon re-encounter with the bacterium. This may enhance local antimicrobial capacity and is associated with a reduced risk of skin reinfection (Hendriks et al., 2021). In systemic infection models, Th17 cells can undergo Th1-like phenotypic conversion with T-bet involvement, thereby contributing to effective bacterial clearance in murine infection models. This demonstrates that dynamic conversion between different Th lineages is a key feature of CD4+ T-cell participation in antimicrobial defense (Bartsch et al., 2022). Compared with the protective responses described above, the functional orientation of CD4+ helper T-cells can shift in inflammatory skin diseases complicated by *S. aureus* infection. In children with atopic dermatitis, Clowry et al. (2024) reported that *S. aureus*-complicated skin lesions exhibit a T-cell profile characterized by co-upregulation of Th2/Th22 and IL-17-related factors, suggesting concurrent adaptive immune activation and inflammatory drive with limited pathogen control efficiency. Humeau et al. (2022) further proposed that keratinocytes form a cytokine interaction network with infiltrating T-cells through TSLP and IL-33, thereby helping to maintain type II inflammation and impaired barrier function. A similar trend was observed in chronic eosinophilic rhinosinusitis with nasal polyps, suggesting that this phenomenon may also occur in the upper airway (Gan et al., 2025). These findings suggest that in *S. aureus*-associated diseases, CD4^+^ helper T-cells can exert protective effects through both TRM and Th17/Th1 responses, while also being associated with persistent inflammation and barrier damage under specific inflammatory conditions. However, the mechanistic evidence primarily stems from animal experimental models.

During chronic or recurrent *S. aureus* infection, CD4+ T-cell function can be constrained by metabolic and immunosuppressive pathways. In chronic infection models, myeloid-derived suppressor cells (MDSCs) enhance their own glycolysis and lactate efflux, thereby limiting lactate transport and NAD+ regeneration in CD4+ T-cells. This suppresses glycolytic activity and effector cytokine production, making MDSCs a major brake on T-cell function during persistent infection (Goldmann and Medina, 2023). Pothlichet et al. (2022) further showed that certain microbial proteins, including components of *S. aureus*, can bind gC1qR and increase PLA2G1B activity at the CD4+ T-cell membrane. This induces an energy-restricted state and is associated with impaired antimicrobial responsiveness. In the vaccine setting, Hajam et al. (2024) showed that long-term colonization by opportunistic pathogens can establish an IL-10-high suppressive imprint in CD4+ T-cells. Upon vaccine restimulation, this imprint weakens IL-17A responses, suggesting that chronic colonization may reduce the efficacy of T-cell-directed vaccines against *S. aureus*.

In addition, virulence factors can further shape CD4+ T-cell responses in a context-dependent manner. In a repeated skin and soft tissue infection model, α-toxin (Hla) impaired dendritic-cell expansion and weakened CD4+ T-cell priming, suggesting that cytotoxin exposure may compromise the quality of subsequent T-cell responses and immune memory (Teymournejad et al., 2023). Recent human *in vitro* studies further suggest that α-toxin affects differentiating helper T-cells not only through impaired priming, but also through direct transcriptional and epigenetic reprogramming. In differentiating human Th17 cells, α-hemolysin altered Th17-associated gene expression and multiple epigenetic regulators, induced changes in histone marks and genome methylation, and was associated with reduced IL-17A, IL-17F, and IL-22 expression (Pastwińska et al., 2024). In a parallel study of differentiating human Th1 cells, α-hemolysin induced widespread DNA methylation changes, particularly at non-CpG sites, together with altered HELLS, DNMT3A, and DNMT3L expression and increased H3K36me3 (Karwaciak et al., 2025). Together, these findings suggest that Hla may affect CD4+ T-cell immunity not only indirectly through impaired antigen-presenting cell support, but also by directly reshaping helper T-cell transcriptional and epigenetic programs. In a humanized MHC II transgenic mouse model of bacteremia, superantigen-driven CD4+ T-cell activation was associated with excessive IFN-γ production, increased visceral colonization, and aggravated disease severity (Tuffs et al., 2022). Consistent with this, Tofacitinib alleviated superantigen-mediated toxic shock by suppressing CD4+ T-cell activation and Th1-related factor expression, further supporting the view that virulence factor–driven CD4+ T-cell responses may shift from host defense toward immunopathology under specific experimental conditions (Jarneborn et al., 2024). Overall, CD4+ helper T-cell responses in *Staphylococcus aureus* infection are highly context-dependent, with protective and pathogenic effects jointly shaped by infection site, disease stage, and the local inflammatory microenvironment. Clarifying these functional states is important for understanding how antimicrobial immunity and immunopathology coexist during infection.

#### 2.2.2 Mechanism of CD4^+^ regulatory T-cells in *Staphylococcus aureus* infection

In acute infections such as *Staphylococcus aureus*-induced septic arthritis and osteomyelitis, CD4^+^ regulatory T-cells (Tregs) act as a classic double-edged sword subset—limiting tissue damage while potentially causing immunosuppression. They primarily influence inflammation severity and bone destruction by regulating the Th17/Treg balance. Multiple studies indicate that interventions such as neutralizing IL-17, supplementing IL-2, and endogenously neutralizing TGF-β and IL-6 can reduce Th17 reactive oxygen species (ROS) levels, enhance Treg levels, improve the RANKL/OPG ratio, and modulate TLR2 and TNFR1/TNFR2 expression, thereby inhibiting bone destruction and oxidative damage. Blocking TGF-β and IL-21 reduces peripheral Th17/Treg ratios to mitigate joint damage. Ghosh et al. (2021, 2022) and Pramanik et al. (2025) proposed that modulating the Th17/Treg balance through different targets could significantly alleviate the progression of diseases such as septic arthritis. At the receptor and signaling pathway level, combined neutralization of TLR2 with TNF-α or IL-1β inhibits STAT3/mTOR and NF-κB activation, promoting Th17-to-Treg conversion. This further supports the role of Tregs in controlling acute inflammation (Ghosh and Bishayi, 2024). Research on natural small molecules suggests this immune axis is pharmacologically modifiable. Berberine downregulates Th17 and Treg-related signaling as well as the JNK/NF-κB-RANKL pathway in *S. aureus*-induced septic arthritis, thereby alleviating inflammation and bone destruction (Asila et al., 2022); Green acid alleviates local immunosuppression and bone damage in a post-traumatic osteomyelitis model by inhibiting IL-2-mediated Treg hyperproliferation (Lin et al., 2025). Combining these findings, the regulatory role of Tregs in acute *S. aureus* infection exhibits a phased pattern: during periods of high inflammatory burden, moderate Treg enhancement helps limit inflammation and tissue damage, whereas in phases dominated by immunosuppression, suppressing excessive Treg expansion better facilitates the restoration of effective antimicrobial responses. The evidence suggests that in experimental models, targeting the Th17/Treg balance using traditional Chinese medicine and its active components may offer a novel immunomodulatory approach with potential translational value for acute infections associated with *S. aureus*.

Unlike their inflammation-limiting role in acute infection, Tregs in chronic, biofilm-associated, mucosal, or specific host contexts are more likely to maintain an immunosuppressive microenvironment that hinders pathogen clearance. Using single-cell sequencing of synovial monocytes in prosthetic joint infection, Wu et al. found marked expansion of monocyte-derived myeloid-derived suppressor cells (M-MDSCs) and Tregs. These cells formed a positive feedback loop through the CXCL16/CXCR6/TGF-β pathway, thereby persistently weakening local antibacterial immunity. This mechanism is considered an important contributor to biofilm persistence (Wu et al., 2025). In respiratory co-infections, Li et al. (2025) reported marked abnormalities in the Th17/Treg balance along the gut-lung axis during H1N1 and MRSA co-infection. Homogeneous polysaccharides from Houttuynia cordata corrected this imbalance by inhibiting complement and NLRP3 inflammasome activation, thereby improving pulmonary pathology. This suggests that modulating the Th17/Treg balance within the mucosal microenvironment may restore anti-infective capacity. Chaihu Guizhi Tang reduced viral and bacterial loads in H1N1 ^+^ *S. aureus* co-infections, downregulated multiple pro-inflammatory cytokines, and restored the Th17/Treg ratio (Han et al., 2025). These studies, examining both localized biofilm and mucosal infection scenarios, collectively indicate that Treg-related Th17/Treg dysregulation is closely associated with persistent inflammation and impaired pathogen clearance. At longer-term developmental and genetic levels, the host baseline immune state may also influence Treg responses to *S. aureus*. Gao et al. (2021) demonstrated that exposure to staphylococcal enterotoxin B during pregnancy reduced FoxP3 methylation in offspring and enhanced Treg number and function, thereby altering subsequent immune-response patterns. At the population level, Clegg et al. observed that *S. aureus*–specific TIGIT^+^ Tregs are widely present in healthy individuals and suppress antigen-induced inflammatory responses *in vitro*. This finding suggests that this subset may attenuate protective T-cell responses induced by *S. aureus* vaccines (Clegg et al., 2025). Overall, in chronic or biofilm-associated infections and in selected host contexts, CD4^+^ Tregs predominantly display features that maintain immunosuppression and support pathogen persistence. Importantly, clinical observations from human inborn errors of immunity further underscore the relevance of the Th17/Treg axis in host defense against *Staphylococcus aureus*. Patients with STAT3-associated hyper-IgE (Job) syndrome frequently experience recurrent cutaneous and pulmonary *S. aureus* infection, a phenotype linked to impaired Th17 differentiation and reduced IL-17/IL-22 production, which in turn compromises epithelial induction of neutrophil-recruiting chemokines and antimicrobial peptides (Park and Liu, 2020; Tsilifis et al., 2021). Achieving precise regulation of this axis across different infection stages and microenvironmental contexts therefore remains an important challenge for future antimicrobial immunotherapy and *S. aureus* vaccine design.

#### 2.2.3 Mechanisms of action of CD8+ cytotoxic T lymphocytes (CTLs) in *Staphylococcus aureus* infection

During *S. aureus* infection, CD8+ cytotoxic T-cell (CTL) responses are highly heterogeneous across tissue microenvironments and are shaped by antigen-presentation pathways, superantigen exposure, and local inflammatory context. Skin infection models suggest that although CTLs can be effectively activated, their role in protection against reinfection is limited. Egawa et al. (2021) found that during *S. aureus* skin infections, bacterial antigens rapidly reach draining lymph nodes and induce antigen-specific CD8+ T-cells, leading to the establishment of skin-resident memory T-cells. However, their protective effect against reinfection is weak, suggesting that locally recruited CTLs are not the major determinant of bacterial control in the skin. In contrast, CTLs in the mucosal environment are more readily recruited to participate in inflammatory and pathological processes under superantigen stimulation. Dufresne et al. (2025) demonstrated in a vaginal colonization model that TSST-1 activates local CD8+ T-cells, induces mucosal inflammation, and alters the microbial community structure, thereby promoting and prolonging vaginal colonization by *S. aureus*. At the human TCR repertoire level, Shepherd et al. (2023) found that Streptococcal Pyrogenic Exotoxin C (SpeC) and Toxic Shock Syndrome Toxin-1 (TSST-1) selectively expand TRBV12-3/12-4+ memory CD4+/CD8+ T-cells and drive high expression of cytokines and co-inhibitory receptors, supporting the view that superantigens can remodel T-cell clonality and functional polarization in a context-dependent manner. Although SpeC is derived from streptococci, this study provides useful insight into TSST-1–related T-cell remodeling through shared superantigen interaction patterns. Mucosal-associated pathology is also observable in intestinal models. Tabiś et al. (2022) reported in an intestinal model that oral administration of SEC/SEL induces T-cell activation characterized by a CD8α cytotoxic phenotype in intestinal epithelial lymphocytes and mesenteric lymph nodes, accompanied by marked villous damage. Taken together, these findings suggest that *S. aureus*-associated CTL responses may exert limited protective effects in skin tissues but are more readily shifted toward inflammatory and barrier-disruptive phenotypes in mucosal settings under sustained superantigen exposure. Their functional orientation is closely linked to antigen properties, TCR preferences, and the tissue microenvironment.

At the mechanistic level, CTL responses in *S. aureus* infection can be either protective or pathogenic, depending on humoral immune status and multiple intracellular regulatory pathways. Miller et al. observed in a post-influenza *S. aureus* pneumonia model that, in the absence of B cells or antibodies, abnormal expansion of pulmonary cytotoxic memory CD8+ T-cells reduced bacterial load but significantly worsened pneumonia and tissue injury. These findings suggest that impaired humoral immunity may amplify CTL-mediated effects and thereby promote immunopathology (Miller et al., 2025). Metabolic pathways are also important regulators of CTL effector function and fate. In an SEA model, nicotinamide reduced IFN-γ and other effector molecules in CD8+ T-cells by inhibiting mTORC1 activity, thereby promoting a memory-like phenotype and alleviating superantigen-induced lung injury. This provides experimental support for targeting rate-limiting steps in CTL effector function through metabolic intervention (Agliano et al., 2022). Ionic homeostasis also contributes to the regulation of cytotoxic activity. Zöphel et al. (2023) identified key molecules, including CCR5, KCNN4, BCL2, and RCAN3, that positively or negatively regulate Ca^2+^-dependent degranulation, thereby defining a molecular network involved in CTL killing-synapse formation beyond the classical STIM/Orai pathway. In parallel, antigen design may be used to preferentially induce protective CTL responses. Yi et al. (2021) showed that immunization strategies targeting secretion system–associated antigens such as EsxA and EsxB can induce protective responses dominated by Th1 and CD8+ T-cells, thereby enhancing defense against *S. aureus*. Overall, CTL responses in *S. aureus* infection are highly plastic, and their protective or damaging effects are jointly shaped by the tissue microenvironment, superantigen exposure, and metabolic or ionic regulation. Future immunological interventions and vaccine design should aim to maximize the antimicrobial potential of CD8+ T-cells while limiting excessive activation through metabolic reprogramming and pathway-specific regulation. Such an approach may help balance antibacterial efficacy with tissue preservation.

#### 2.2.4 Mechanisms of γδ T cells and innate-like T cells (NKT, MAIT) in *Staphylococcus aureus* infection

During *Staphylococcus aureus* infection, γδ T cells and other “innate-like” T cells play a crucial role in early immunity, with their core pathways predominantly associated with IL-17. Research indicates that the diverse disease progression triggered by *S. aureus*—ranging from localized skin infections to systemic sepsis–relies on the effective activation of IL-17-related pathways (Bekeredjian-Ding et al., 2015). γδ T cells are regarded as a crucial cell population bridging innate and adaptive immunity, as they can rapidly produce effector molecules without requiring classical antigen presentation. They can be subdivided into functional subsets primarily characterized by IFN-γ and IL-17A production (Nanda and Alphonse, 2024). In a mouse model of systemic MRSA infection, genetic evidence indicates that T-cell-intrinsic IL-1R signaling is more critical for survival than direct deletion of IL-17A/F or TNF-α, and γδ T cells are the primary source of IL-17A dependent on IL-1R (Wang et al., 2023). Experimental evidence suggests that the IL-1R-γδT-IL-17 pathway contributes to limiting early bacteremia. At the level of barrier tissues, IL-17-producing γδ T cells participate in neutrophil recruitment and clearance of *S. aureus* in the mammary gland, skin, and respiratory tract. Pan et al. (2023) found in a mastitis model that Vγ4^+^ γδ T cells serve as the primary source of IL-17A during early infection. Their activation depends on the local microbiota, thereby promoting neutrophil recruitment and bacterial clearance. Within the skin, Park et al. (2022) reported that microbiome-induced CD169^+^ macrophages maintain IL-17-producing γδ T cells activity via type I interferon and IL-23 signaling. When this pathway is impaired, the host skin becomes more susceptible to *S. aureus*. In the lungs, Zhang et al. (2025) demonstrated that commensal Lactobacillus enhances the IL-17A response of Vγ4^+^ γδ T cells, thereby improving host resistance to *S. aureus* and Pseudomonas aeruginosa pneumonia. Collectively, these studies support the existence of a microbiota-regulated γδ T cells-neutrophil defense pathway centered on IL-1R/IL-17A across multiple organ barriers, which plays a crucial role in the early clearance of *S. aureus*. Consistent with this view, recent spatial transcriptomic work in human barrier-tissue inflammation shows that γδ T cells can occupy defined tissue niches with sentinel-like programs, yet progressively acquire exhaustion-associated transcriptional features in chronic or highly inflammatory microenvironments—an observation that may help interpret γδ T-cell dysfunction during persistent or recurrent *S. aureus* barrier infections (Bonilha, 2026).

In humans, γδ T cells and other “innate-like” T cells, such as NKT and MAIT cells, participate in the response to *S. aureus* through multiple non-classical recognition pathways. These cells can mediate acute antimicrobial protection, but may also contribute to immune evasion and chronic inflammation. With respect to human Vγ9Vδ2 cells, Eberl et al. (2025) noted that although *S. aureus* does not synthesize classical phosphoantigens such as HMB-PP, it can indirectly activate this population by altering BTN2A1/BTN3A1 conformation, inducing host metabolic stress, and secreting toxins and superantigens. Uzunçayir et al. (2025) further showed that the superantigen SEA can directly bind the Vγ9Vδ2 TCR and activate γδ T cells with only moderate MHC II dependence, suggesting a recognition pattern distinct from that of classical αβ T cells. For human type II NKT cells, Genardi et al. (2020) demonstrated that these cells recognize MRSA-derived polar lipid antigens via CD1d and produce IFN-γ, thereby reducing bacterial burden in systemic infection. MAIT cells recognize bacterial vitamin B metabolites via MR1. Cooper et al. (2022) observed that, following dendritic-cell presentation of *S. aureus*, human MAIT cells rapidly produce IFN-γ and granzyme B, thereby reducing intracellular bacterial survival. At the same time, these innate-like T-cell populations may also become targets of pathogen-mediated immune evasion and drivers of chronic inflammatory amplification. In human cell-based analyses, MAIT cells were found to be highly sensitive to LukED produced by *S. aureus*. Specifically, CCR5-high MAIT cells and mature CD57+ NK cells were more susceptible to selective lysis, suggesting that leukotoxin-mediated depletion of innate-like effector populations may weaken mucosal immune surveillance and facilitate pathogen immune evasion at barrier sites (Boulouis et al., 2022). In chronic skin lesions, Jamet et al. (2025) observed in pediatric patients with recessive dystrophic epidermolysis bullosa that highly inflammatory *S. aureus* strains adapted to the skin environment can induce an increased proportion of IL-17A+ MAIT/Th17 cells, which correlates with disease severity. These findings suggest that innate-like T cells may also contribute to sustained regulation of chronic inflammation and tissue injury. Collectively, γδ T cells and innate-like T cells such as NKT and MAIT cells form a cross-tissue immune network that frequently relies on IL-17-dependent barrier defense, while also showing context-dependent contributions to systemic control, chronic inflammation, and immune evasion. In experimental studies and *in vitro* systems, this network can support early antibacterial defense, but under sustained toxin exposure or chronic inflammatory conditions it may also participate in barrier disruption, persistent inflammation, or loss of protective effector populations (Genardi et al., 2020; Boulouis et al., 2022; Cooper et al., 2022; Nanda and Alphonse, 2024; Eberl et al., 2025; Zhang et al., 2025).

## 3 Context-dependent T-cell dysregulation: immunopathology, immune evasion, and the Th17/Treg axis

### 3.1 Immunopathology mediated by T-cell responses

In *Staphylococcus aureus* infection, T-cells can contribute to immunopathology while still participating in pathogen control. Current evidence suggests that T-cell–related immunopathology can be understood along three major but interconnected dimensions: superantigen-driven effector amplification, toxin-mediated impairment of protective immune memory, and mucosal immune skewing toward pathogenic inflammation. First, superantigens can drive excessive and nonphysiologic T-cell activation rather than uniformly protective immunity. By bridging MHC II and specific TCR Vβ chains, *S. aureus* superantigens amplify selected CD4^+^ T-cell populations and promote marked inflammatory cytokine production. In a humanized MHC II transgenic mouse model of bacteremia, Tuffs et al. (2022) showed that IFN-γ-dominant T-cell responses were associated with increased hepatic bacterial burden and mortality. In chronic inflammatory human skin lesions, Ortega Rocha et al. (2024) found that deposition of *S. aureus* and its enterotoxin B (staphylococcal enterotoxin B, SEB) was closely associated with infiltration of Vβ17^+^ IL-17^+^ CD4^+^ T-cells, and that this population correlated with disease severity and poor response to biologic therapy. Taken together, these findings indicate that superantigen-related T-cell pathology is highly context-dependent: IFN-γ-dominant immunopathology is more evident in invasive experimental settings, whereas IL-17-skewed inflammatory amplification is more prominent in chronic inflammatory tissue lesions. Second, toxins can interfere with the establishment of subsequent protective immunity at the level of immune memory. Teymournejad et al. (2023) showed in a repeated skin and soft tissue infection model that α-toxin expression during primary infection impaired dendritic-cell expansion and reduced T-cell responses induced by the inactivated toxin vaccine HlaH35L, thereby weakening protective efficacy. This suggests that toxin exposure may influence not only acute inflammation but also the quality of subsequent T-cell memory responses. Third, in mucosal tissues–particularly the upper airway–*S. aureus* toxins may promote immune deviation toward T2-type inflammation and IgE-associated pathology. Bachert et al. (2021); Shah and Kobayashi (2023) reported that *S. aureus* enterotoxins and their specific IgE responses are associated with polyclonal B-cell activation, eosinophil infiltration, and enhanced T2-type inflammation, thereby contributing to the development and recurrence of chronic rhinosinusitis with nasal polyps. Notably, this toxin-related immune imbalance may be at least partially modifiable. Kleinhenz et al. (2024) reported in a pediatric cohort that individuals who developed high-titer, broad-spectrum toxin-neutralizing antibodies during convalescence showed more complete restoration of dendritic-cell and T-cell function. This observation suggests that toxin neutralization may not only reduce pathogen burden but also help limit subsequent inflammatory skewing by reducing direct toxin-mediated immune injury. Overall, current evidence indicates that T-cell–mediated immunopathology in *S. aureus* infection is not a single process, but rather reflects the combined effects of excessive effector amplification, impaired memory formation, and tissue-specific immune deviation. Accordingly, the toxin–T-cell axis, including superantigens and cytotoxins, represents an important mechanistic basis for immunopathology and a potential target for vaccine design and antitoxin immunotherapy. Importantly, several of these observations derive from local tissue lesions, experimental models, or cell-based systems and therefore should not be directly extrapolated to peripheral blood immune profiles in human infection.

Virulence factors can also shape T-cell memory through distinct mechanisms. Hla impairs dendritic-cell expansion and weakens vaccine-elicited T-cell memory in repeated skin and soft tissue infection models, while recent human *in vitro* studies further suggest that it can reprogram differentiating Th17 and Th1 cells at transcriptional and epigenetic levels (Teymournejad et al., 2023; Pastwińska et al., 2024; Karwaciak et al., 2025). Superantigens, by contrast, remodel TCR usage and selectively expand TRBV12-3/12-4^+^ memory CD4^+^/CD8^+^ T-cell populations, supporting biased repertoire imprinting rather than uniformly protective recall responses (Shepherd et al., 2023). In barrier settings, leukotoxin-mediated depletion of MAIT cells and mature NK cells may further weaken local recall protection by reducing innate-like effector populations that support mucosal surveillance (Boulouis et al., 2022). Together, these findings suggest that virulence factors act at distinct nodes—including antigen-presenting cell support, repertoire selection, and barrier effector maintenance—to influence the quality of T-cell memory and local protective immunity.

### 3.2 *Staphylococcus aureus*–induced T-cell immune escape

During *Staphylococcus aureus* infection, T-cells–related immune escape does not arise from a single mechanism but rather reflects a multilayered regulatory process driven by persistent antigen exposure and the inflammatory microenvironment. As infection progresses from acute to chronic stages, the pathogen reshapes the metabolic landscape, perturbs effector and memory T-cell fate, compromises T-cell signaling and antigen-presentation quality, and induces humoral immune bias. Together, these changes progressively constrain protective T-cell responses and may contribute to long-term colonization or recurrent infection (Teymournejad and Montgomery, 2021). Importantly, this process is not linear, but reflects the coordinated action of multiple suppressive checkpoints operating across temporal and spatial scales. At the metabolic level, chronic infection models show that expanded MDSCs can suppress CD4^+^ T-cell glycolysis and effector molecule production by enhancing their own glycolysis and lactate efflux, thereby promoting a state of metabolic exhaustion. Goldmann and Medina (2023) suggested that lactate metabolism plays an inhibitory role in this process and may help support long-term bacterial colonization. In this chronic infection model, the suppressive metabolic program is attributed mainly to expanded MDSCs, whereas the downstream readouts include reduced glycolytic activity and diminished effector molecule production in CD4^+^ T-cells (Goldmann and Medina, 2023). In a tissue-localized biofilm setting, single-cell analysis of prosthetic joint infection further identified expansion of M-MDSCs and Tregs within the infected synovial microenvironment and linked them through a CXCL16/CXCR6/TGF-β feedback loop, supporting the existence of a localized suppressive circuit in biofilm-associated disease (Wu et al., 2025). With respect to memory T-cell fate, Deng et al. (2021) reported that adenosine synthase A secreted by *S. aureus* under experimental conditions reduces IL-1β-driven Th17 polarization through the adenosine A2a receptor–NLRP3 pathway, thereby potentially attenuating IL-17A-dependent protective immunity; Hajam et al. further showed that prior infection or commensal bacteria can induce IL-10^+^CD4^+^ memory T-cells specific for *Staphylococcus aureus*. This population is preferentially recalled during vaccine re-stimulation and suppresses IL-17A production, which may influence the quality of the memory response (Hajam et al., 2024). At the interface between humoral bias and T-cell immune escape, *Staphylococcus aureus* protein A (SpA) warrants particular attention. In human infection studies, SpA acts as a B-cell superantigen that biases plasmablast responses toward SpA itself while limiting effective recognition of other protective antigens (Pauli et al., 2014). This bias is relevant to T-cell immunity because it may narrow the breadth of antigen available for productive uptake and presentation. Consistent with this, experimental evidence indicates that the SpA–peptidoglycan complex can promote non-protective VH3-biased B-cell activation and alter antigen-clearance kinetics and antigen-presentation quality (Shi et al., 2021). Although largely indirect, these upstream effects may compromise downstream T-cell priming and impair the quality of protective memory responses. Thus, the contribution of SpA to T-cell immune escape should not be viewed solely as a humoral phenomenon, but also as an upstream determinant of antigen availability and presentation. Additional immune-evasion mechanisms may further constrain protective T-cell responses at the levels of signaling perception and antigen presentation. Pothlichet et al. (2022) showed that certain microbial proteins can bind gC1qR and enhance PLA2G1B-dependent remodeling of CD4^+^ T-cell membrane microarchitecture, thereby promoting an unresponsive phenotype. Although this mechanism has not been fully validated for specific *S. aureus* proteins, it suggests that pathogen-induced receptor-level perturbation may dampen T-cell responsiveness under some infectious conditions. At the level of antigen presentation, experimental evidence further indicates that systemic bacterial infection can promote biased hematopoiesis, impair dendritic-cell precursor development, and reduce MHC II expression, which may lead to chronic dendritic-cell deficiency and subsequent weakening of T-cell responses (Bieber et al., 2021). In experimental systems, *S. aureus* has been shown to suppress protective T-cell immunity through multiple immune-evasion strategies, which may help explain the impaired T-cell responses observed in human infection. Collectively, these mechanisms form a complex network of T-cell immune escape and may provide mechanistic insight into the limited protective efficacy observed in clinical trials of several *Staphylococcus aureus* vaccines (Tsai et al., 2022).

Direct evidence for classical checkpoint pathways in *S. aureus* T-cell immune evasion remains limited. Current *S. aureus*-specific findings are largely observational or indirect, including the presence of *S. aureus*-specific TIGIT^+^ Tregs in healthy individuals and inhibitory-receptor–associated T-cell dysfunction in children with invasive infection (Li et al., 2022; Clegg et al., 2025). By contrast, broader implications of PD-1/PD-L1 or CTLA-4 signaling for *S. aureus* immune evasion are still mainly extrapolated from age-stratified antigen-specific T-cell models outside direct *S. aureus* settings (Arra et al., 2021; Majer et al., 2023). This distinction should be kept explicit when discussing checkpoint-related signaling nodes in *S. aureus* infection.

### 3.3 Context-*dependent dysregulation* of the Th17/Treg *axis* in *Staphylococcus aureus infection*

The Th17/Treg axis represents an important but context-dependent regulatory module in *Staphylococcus aureus* infection rather than a universal driver across all disease settings. In barrier-tissue infection, chronic inflammation, and biofilm-associated persistence, shifts between IL-17-dominant effector responses and Treg-associated suppressive programs can strongly influence the balance between bacterial control and inflammatory damage. However, in invasive disease and bacteremia, Th1/IFN-γ responses and superantigen-driven immunopathology may be more dominant, whereas in implant- or biofilm-associated infection, physical barriers, tolerogenic myeloid programs, dendritic-cell impairment, and metabolic restriction may outweigh a classical Th17/Treg framework. Accordingly, the Th17/Treg axis is best interpreted as one major component of context-specific immune regulation rather than a single overarching explanation for all *S. aureus* syndromes. This view is also consistent with prior reviews emphasizing that Th17- and Treg-associated programs operate within a narrow and context-sensitive functional window (Armentrout et al., 2020; Teymournejad and Montgomery, 2021). In chronic infection settings, this axis often shifts toward an inhibitory state. Goldmann and Medina (2023) reported that expanded MDSCs in chronic *S. aureus* infection suppress CD4+ T-cell glycolysis and effector molecule production through lactate metabolism, thereby weakening Th17-associated antimicrobial function and promoting a Treg-dominated suppressive microenvironment; In chronic airway inflammatory conditions such as cystic fibrosis, persistent accumulation of granulocytic MDSCs has likewise been associated with an immunosuppressive phenotype, and Tucker et al. (2021) suggested that similar mechanisms may operate in selected chronic *S. aureus*–associated settings. When infection progresses to systemic inflammation or sepsis, broader metabolic dysfunction may become more important than a classical Th17/Treg framework alone. In a *S. aureus* sepsis model, Reizine et al. (2022) showed that arginine deficiency and mitochondrial dysfunction limited T-cell proliferation, whereas enteral citrulline supplementation restored mitochondrial metabolism, enhanced proliferation, and reduced mortality from secondary infections. These findings suggest that, in invasive disease, metabolic support of T-cell fitness may be more immediately relevant than viewing outcome primarily through the Th17/Treg axis alone. During early life, immune checkpoint–mediated inhibition may also influence establishment of the Th17/Treg axis. Majer et al. and Arra et al. independently confirmed that PD-1/PD-L1 and CTLA-4 pathways exhibit more pronounced inhibitory effects in antigen-specific CD4^+^ T-cells from neonates and children, limiting full T-cell activation and differentiation. This may consequently influence Th17-related memory formation (Arra et al., 2021; Majer et al., 2023). Consistent with this, Li et al. (2022) reported that children with invasive *S. aureus* infections exhibited weakened systemic and antigen-specific T-cell responses during the acute phase, with incomplete recovery in some individuals after convalescence. These data support the existence of durable immune dysfunction after invasive infection, but do not by themselves establish the Th17/Treg axis as the dominant explanatory framework in this setting. By contrast, the microbiome may modulate this axis through a more physiological route. Vogel et al. found that commensal Bifidobacterium induces FoxP3^+^ Tregs, IL-10, and galectin-1 production, thereby suppressing excessive Th1/Th17 responses against *S. aureus* to buffer inflammation. However, its impact on *S. aureus* clearance efficiency requires further evaluation (Vogel et al., 2023). In summary, the Th17/Treg axis is susceptible to bidirectional dysregulation influenced by metabolic status, developmental stage, and microecological factors: Treg dominance may lead to inadequate clearance and chronic colonization, while excessive Th17 activity may exacerbate inflammatory damage. This provides immunological rationale for developing precision immunotherapy strategies. These scenario-dependent distinctions are further summarized by clinical phenotype in Table 2.

**Table 2:** Context-dependent dominant immune programs across major clinical phenotypes of *Staphylococcus aureus* infection.

Clinical phenotype	Dominant immune program	Key virulence factors/contextual drivers	Therapeutic implication
Skin and soft tissue infection/recurrent cutaneous infection	Early barrier-protective Th17, γδT and TRM responses; in chronic lesions, mixed inflammatory amplification may emerge	Hla, local barrier disruption, microbiota shifts	Enhance barrier immunity and tissue-resident memory while preventing persistent inflammatory amplification
Invasive disease/bacteremia/toxic shock	Th1/IFN-γ-dominant responses and superantigen-driven immunopathology	TSST-1, enterotoxins, systemic inflammatory amplification	Anti-toxin strategies, control of excessive inflammation, and context-specific immunomodulation
Post-influenza secondary bacterial pneumonia	Lung TRM/Th1 support with risk of excessive CD8-mediated tissue injury	Viral-bacterial inflammatory synergy, cytotoxins, impaired humoral control	Preserve protective lung memory while limiting immunopathology
Biofilm/implant-associated infection	Treg/MDSC expansion, tolerogenic myeloid programs, dendritic-cell impairment, and metabolic restriction	Biofilm barrier, CXCL16/CXCR6/TGF-β loop, lactate-mediated suppression, impaired antigen presentation	Combine biofilm control with reversal of suppressive myeloid and metabolic programs
Nasal colonization/chronic carriage	Restricted IL-17/IL-22 responses with increased IL-10/IL-27 and incomplete local protective immunity	Enterotoxins, inhibitory cytokine milieu, mucosal immune bias	Strengthen mucosal immunity and local memory rather than relying on systemic immunogenicity alone
Chronic rhinosinusitis with nasal polyps/T2-skewed mucosal disease	T2/IgE-biased inflammation rather than classical Th17/Treg dominance	Enterotoxins, IgE-associated amplification, eosinophilic inflammation	Anti-toxin plus endotype-specific anti-inflammatory intervention

## 4 T-cell responses and *Staphylococcus aureus*-related diseases and prevention strategies

### 4.1 Overall characteristics of T-cell responses in related diseases and special populations

*Staphylococcus aureus* causes a wide range of clinical syndromes, including skin and soft tissue infection, pneumonia, bacteremia, nasal colonization, and postoperative biofilm-associated infection. Although these conditions all involve T-cell immunity, they are not governed by a single dominant immune program. Rather, each clinical phenotype reflects a distinct balance among barrier-protective Th17/TRM responses, Th1/IFN-γ-dominant invasive control, superantigen-mediated immunopathology, tolerogenic myeloid suppression, and biofilm-related metabolic or antigen-presentation constraints. This context dependence is critical for interpreting immune correlates of protection and for designing targeted prevention or treatment strategies. To make these context-dependent distinctions more explicit across major disease settings, Table 2 summarizes the dominant immune programs, key virulence factors or contextual drivers, and corresponding therapeutic implications associated with the major clinical phenotypes of *S. aureus* infection.

Within this framework, the studies below illustrate how distinct protective or pathogenic T-cell programs emerge in specific clinical or experimental settings rather than uniformly across all *S. aureus* disease phenotypes. Santos et al. (2021) found in breast and systemic infection models that specific antigens associated with protection can induce IL-17A-rich type III memory T-cells. The abundance of these cells correlates with reduced bacterial load and protective outcomes upon re-challenge, suggesting that Th17-like memory T-cells may help maintain anti-infective immunity in experimental models. In barrier tissues, Hendriks et al. (2021) demonstrated that healthy human skin harbors a substantial population of *S. aureus*-specific CD4^+^ tissue-resident memory T-cells. Braverman et al. (2022) further revealed that *S. aureus*-specific Th1-like TRM in the lungs mitigate the severity of post-influenza secondary bacterial pneumonia. In addition, human-derived MAIT cells and type II NKT cells respond rapidly to *S. aureus* stimulation and produce effector molecules such as IFN-γ. They contribute to early control of intracellular bacterial burden and systemic infection, thereby providing synergistic support for protective T-cell immunity in selected contexts (Genardi et al., 2020; Cooper et al., 2022).

However, in children and in selected populations with chronic inflammatory disease, *S. aureus*–associated T-cell responses may diverge from the protective pattern described above. Li et al. observed in children with invasive *S. aureus* infections that peripheral blood *S. aureus*-specific CD4^+^ and CD8^+^ T-cells exhibited markedly reduced IL-17A and IFN-γ production upon re-stimulation, accompanied by upregulation of inhibitory receptor expression. This suggests impaired T-cell function may increase the risk of persistent bacteremia and recurrence (Li et al., 2022). In populations with chronic nasal carriage of *S. aureus*, observational studies have shown elevated local IL-10 and IL-27 levels in the nasal mucosa together with a restricted IL-17/IL-22 response. In corresponding mouse models, Kelly et al. (2022) showed that blockade of either IL-10 or IL-27 signaling accelerates clearance of colonizing bacteria, suggesting that this inhibitory cytokine axis plays an important regulatory role in maintaining *S. aureus* colonization. In chronic rhinosinusitis with nasal polyps, a condition often characterized by T2-dominant pathogenesis, mucosal tissues show elevated Th2-cell activity and increased IL-4, IL-5, and IL-13 levels, together with local IgE accumulation and eosinophilic infiltration. *Staphylococcus aureus* and its enterotoxins have been implicated as key environmental drivers of polyclonal T-cell activation and inflammatory amplification in this setting (Shah and Kobayashi, 2023). Additionally, among patients with inflammatory skin diseases, Ortega Rocha et al. observed that abnormal distribution of epidermal barrier proteins within skin lesions, alterations in skin microbiota composition (including *S. aureus* and its toxins), and IL-17-biased T-cell infiltration were closely associated with specific TCR clonal expansion. These findings correlated with disease severity and response to biologic therapy (Ortega Rocha et al., 2024). Overall, *S. aureus*–associated T-cell responses appear to span a continuum ranging from protective programs dominated by Th1/Th17 responses and tissue-resident memory T-cells to non-protective states characterized by immunosuppression or dysregulated inflammatory amplification. Together, observational human studies and experimental findings help define this immune continuum and provide an immunological basis for stratified approaches to prevention and management across different disease settings and host contexts (Armentrout et al., 2020; Santos et al., 2021; Teymournejad and Montgomery, 2021).

### 4.2 Overview of T-cell response-based prevention and treatment strategies

Current evidence suggests that prevention of *Staphylococcus aureus* infection should not rely solely on induction of high-titer antibodies. Instead, greater emphasis should be placed on shaping T-cell responses with clear protective relevance. Particularly informative readouts include the multifunctionality of Th1/Th17 effector responses, the persistence of memory T-cells, and the establishment of local immune memory in barrier or infection-related tissues. In chronic or recurrent infection settings, the coordinated induction of IFN-γ^+^ Th1 and IL-17^+^ Th17 responses together with durable memory appears especially important for limiting dissemination and reducing recurrence (Armentrout et al., 2020; Santos et al., 2021; Teymournejad and Montgomery, 2021). Within this framework, one major strategy is the precise selection of protective antigens and epitopes. Santos et al. (2021) discovered in systemic and mammary infection models that specific antigens associated with protective outcomes preferentially induce IL-17A-rich type III memory T-cells during antigen design. Their levels correlate with reduced bacterial load and protection against re-challenge, suggesting that antigens themselves play a crucial role in shaping Th17-like memory. Beyond antigen design, modulation of immunosuppressive pathways has been shown to further enhance T-cell response quality. Kelly et al. demonstrated that transiently suppressing IL-10 signaling during immune processes amplifies Th17/Th22 responses without significantly disrupting overall immune homeostasis, while improving bacterial clearance in systemic and cutaneous infection models. This indicates that precise intervention in inhibitory axes may unlock the potential of protective T-cells (Kelly et al., 2024). However, the transition from candidate identification to clinically meaningful protection remains a major challenge for T-cell-oriented vaccine design.

Immunoinformatics and reverse vaccinology are therefore best interpreted as candidate-prioritization tools rather than direct evidence of protective efficacy. For example, Francis et al. (2021) identified predicted CD4^+^ T-cell epitopes from the *S. aureus* secretome through HLA class II binding prediction and molecular docking, whereas multi-epitope studies such as those by Kolla et al. (2021); Mahapatra et al. (2023) mainly provide predicted antigenicity, HLA coverage, or early preclinical immunogenicity signals rather than direct proof of reduced colonization, prevention of reinfection, or attenuation of disease severity. At the platform level, peptide vaccines remain attractive because of their design flexibility and compatibility with adjuvants or delivery systems, but their translational value still depends on whether predicted or experimental immunogenicity can be matched to clinically relevant protection endpoints (Tavassoli Razavi et al., 2025). The NDV-3A trial provides a useful example of this translational gap. In that phase 2 study, the relevant endpoint was prevention of *S. aureus* acquisition/colonization rather than broader disease-severity outcomes, and a single dose of NDV-3A did not prevent nasal or oral acquisition despite encouraging immunogenicity findings (Millar et al., 2021). This suggests that systemic immunogenicity alone is insufficient to predict success at a specific protection endpoint. It also highlights that colonization control depends on factors beyond systemic T-cell activation, including local antibodies, innate immune tone, microbiota interactions, and the establishment of effective mucosal or tissue-resident memory responses. Accordingly, evaluation of immunoinformatics-derived or reverse-vaccinology-based candidates should specify whether the intended protection endpoint is reduced colonization, prevention of recurrent infection, attenuation of disease severity, or mitigation of toxin-mediated immunopathology.

Accordingly, increasing attention has been directed toward how delivery pathways and the local immune microenvironment shape the quality and spatial localization of T-cell responses. Super et al. (2021) used biomaterials to capture pathogen-associated molecular patterns at sites of infection or elevated risk, thereby enabling sustained antigen presentation and dendritic-cell recruitment and enhancing T-cell–mediated protective immunity; Su et al. (2024) further showed that improving the microenvironment at the infection site and activating innate immune signaling pathways such as cGAS-STING can synergistically enhance both innate and adaptive T-cell responses and promote local immune-memory formation. Overall, T-cell–based strategies for preventing and treating *S. aureus* infection are increasingly integrating multiple dimensions, including precise antigen and epitope design, modulation of immunosuppressive pathways, and optimization of delivery routes and local microenvironments. Together, these approaches outline an immunological engineering framework centered on T-cell functional quality and tissue localization. However, its efficacy and safety across different populations and key clinical endpoints require further validation.

Despite progress in delineating T-cell responses during *Staphylococcus aureus* infection, several important limitations remain. Much mechanistic evidence still derives from animal models or *in vitro* systems, and extrapolation to human infection–particularly within complex skin and mucosal microenvironments—requires caution. Clinically, many studies rely on peripheral blood readouts, whereas circulating signatures may not capture immune events within infection foci or tissue-resident compartments (e.g., TRM), thereby limiting cross-study comparability and the stability of proposed immune correlates. In addition, *S. aureus* virulence factors and biofilm-associated immune modulation contribute to pronounced host- and niche-dependent heterogeneity, complicating identification of universal correlates of protection or vaccine targets. Longitudinal human studies that integrate tissue context and disease stage are therefore needed to resolve the dynamic balance among protective immunity, immunopathology, and immune evasion and to facilitate translation. These challenges highlight the need for stratified or context-specific T-cell–targeted strategies that explicitly incorporate patient heterogeneity, tissue localization, and disease stage, thereby improving the adaptability and translational relevance of immune interventions across diverse clinical settings.

## 5 Summary and outlook

Recent studies of T-cell responses in *Staphylococcus aureus* infection have substantially improved our understanding of the dynamic balance among host defense, immunopathology, and immune evasion. This review has focused on T-cell biology from the perspectives of differentiation, recognition, and functional regulation. It has integrated evidence on cytokine networks, immunometabolism, and immune checkpoint regulation to compare the distinct roles of Th1/Th17, Treg, CD8+ T-cells, γδ T cells, and MAIT/NKT populations across different *S. aureus* infection settings. Across skin and soft tissue infection, respiratory disease, bacteremia, biofilm-associated infection, pediatric infection, nasal colonization, and immunocompromised host settings, the available evidence consistently supports a framework in which T-cell responses must be interpreted in terms of protection, immunopathology, immune evasion, and translational intervention.

Current evidence indicates that T-cell responses to *S. aureus* are not uniform, but instead shift across a continuum of functional states. At one end of this continuum are Th1/Th17-dominant and tissue-localized memory responses associated with pathogen clearance. At the other end are virulence factor–driven, especially superantigen-driven, states of excessive activation accompanied by marked immunopathology. Animal models and clinical observations together suggest that dysregulation of the Th17/Treg balance, abnormal IL-1/IL-10 regulation, MDSC expansion, and enhanced immune checkpoint signaling are closely linked to progression from acute inflammation to chronic persistence and biofilm-associated refractory infection. Furthermore, *S. aureus* can impair the durability and coordination of effector responses through metabolic restriction, signaling remodeling, inhibitory cytokine environments, and the induction of biased or exhaustion-like memory T-cell states. These changes may also compromise humoral immune quality and limit long-term vaccine-induced protection. However, much of the current evidence still derives from specific animal models or small human cohorts. Spatiotemporal integration of T-cell networks across different barrier sites, disease stages, drug-resistance backgrounds, and co-infection settings remains limited. In particular, the relationship between systemic immune markers and local barrier immunity, especially TRM-associated protection, requires further clarification.

Looking ahead, basic research must deepen mechanistic integration across three dimensions: effector cell quality, tissue localization, and microenvironmental constraints. On the one hand, single-cell and spatial omics should be used to clarify the interaction networks linking Th17/Treg cells, γδ T cells, and innate-like T-cell populations with neutrophils and dendritic cells, thereby identifying the cellular states most closely associated with pathogen clearance across different stages and tissue sites. On the other hand, integration of infection-related thymic changes, TCR repertoire diversity, peripheral immune metabolism, and immune checkpoint signaling may help identify more reliable biomarkers of protective T-cell immunity. For translational application, antigen selection informed by immune profiles from clinically cured individuals or high-exposure/low-incidence populations may help optimize B-cell and T-cell epitope combinations and improve multi-epitope vaccine platforms. Such an approach may enhance Th1/Th17-associated protection while avoiding excessive immunosuppression. Greater emphasis should also be placed on establishing local immunity and TRM at mucosal and barrier sites, together with stratified evaluation of systemic immunogenicity against clinical endpoints such as colonization and recurrence. Together with precise modulation of immunosuppressive pathways and optimization of delivery strategies and local microenvironments, these advances may help create immune conditions that favor antigen presentation and tissue-resident memory maintenance at infection sites or other high-risk locations. If safety can be established, such strategies may shift anti-*S. aureus* prevention and treatment beyond simple enhancement of antibody responses toward broader immune regulation centered on T-cells.

## Data Availability

No original datasets were generated or analyzed in this study. The data discussed in this article are derived from previously published studies.
